# Supplementary figures and images for: Impairment of lipid homeostasis causes lysosomal accumulation of endogenous protein aggregates through ESCRT disruption
Source: eLife. 2024 Dec 23;12:RP86194. doi: 10.7554/eLife.86194 (PMC11666243; doi:10.7554/eLife.86194)

Figure 3 – source data 2

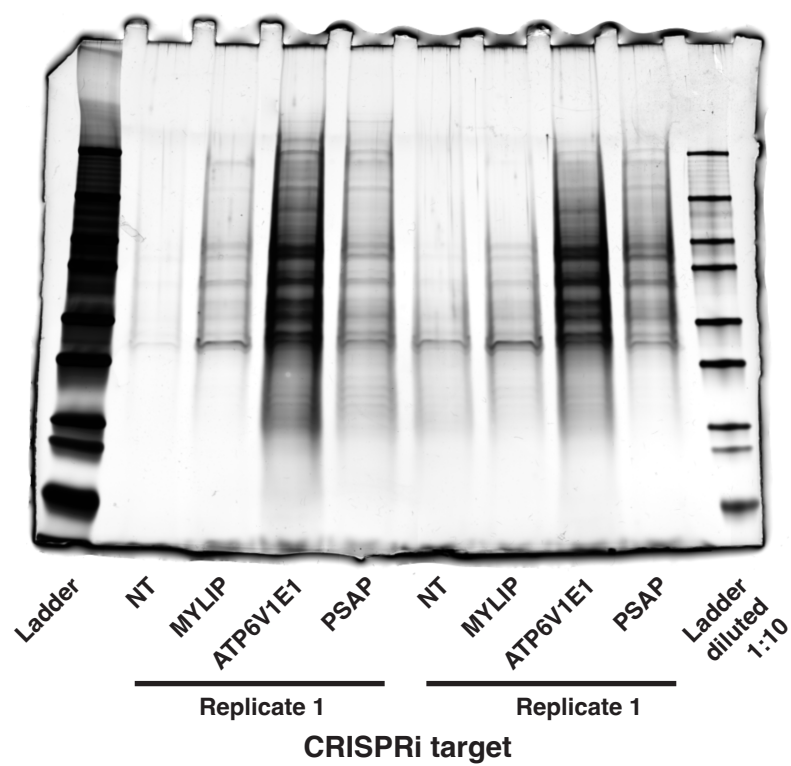

Ladder = BioRad Precision Plus Dual Color Protein Standards

Supplement: Figure 3—source data 1. [file elife-86194-fig3-data1.pdf]

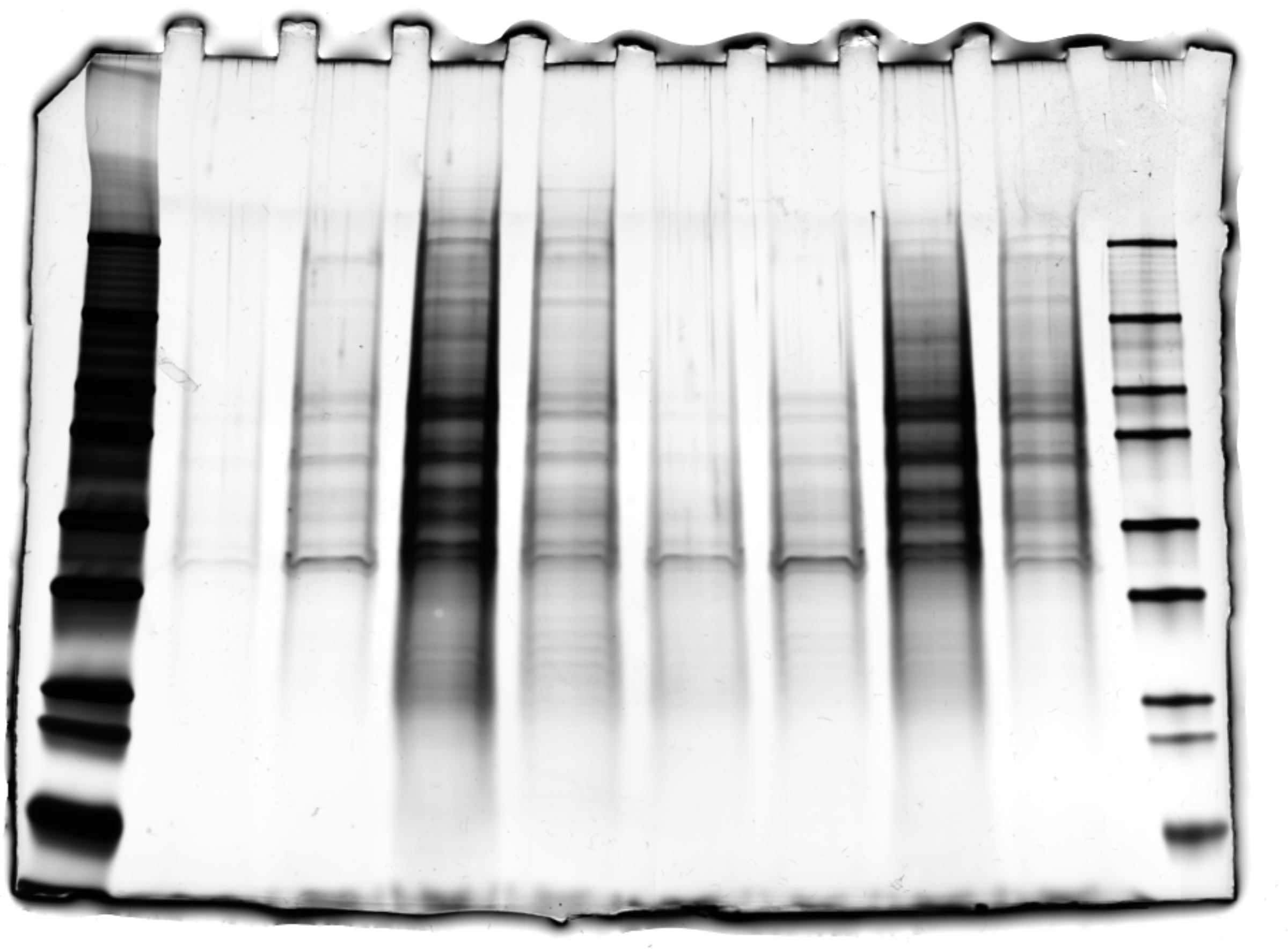

Supplement: Figure 3—source data 2. [file elife-86194-fig3-data2.zip › Figure 3-source data 2/CRISPRi_LysoIP_UreaSDS_Silverstain_raw.tif]

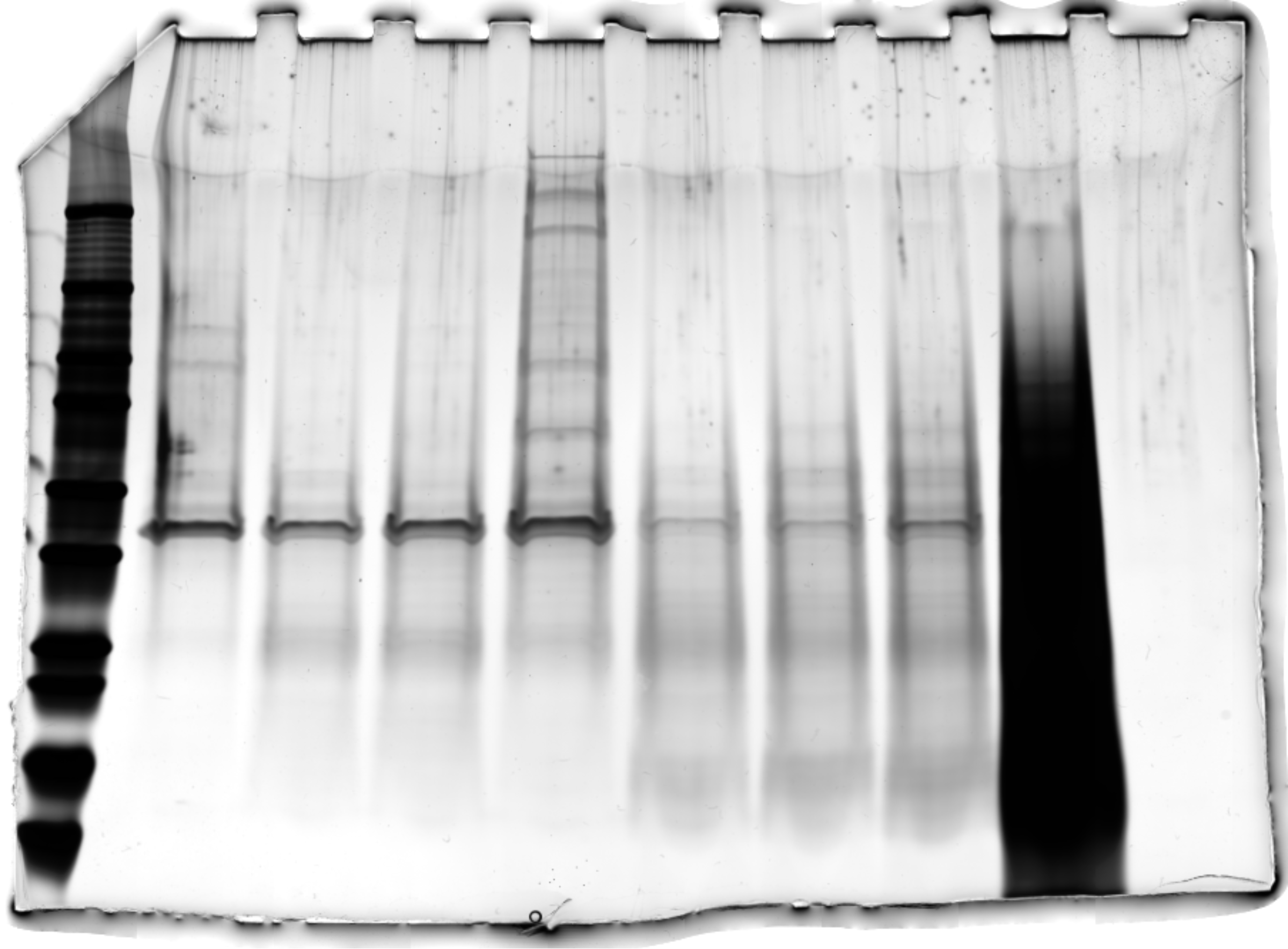

Supplement: Figure 3—figure supplement 2—source data 2. [file elife-86194-fig3-figsupp2-data2.zip › Figure 3-figure supplement 2-source data 2/MG132_series_Mock_and_Lyso-IP_Silverstain_raw.tif]

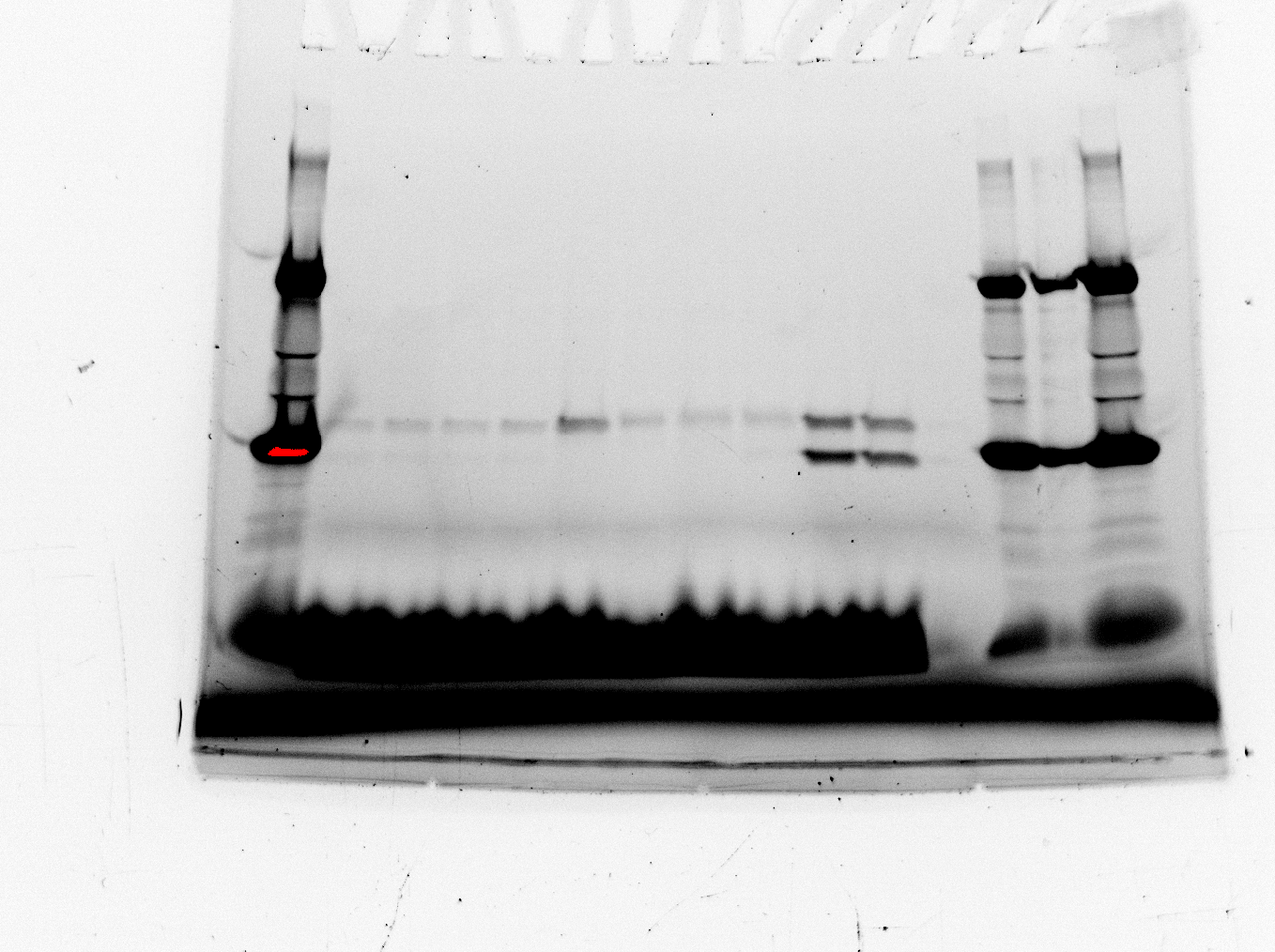

Supplement: Figure 6—figure supplement 1—source data 2. [file elife-86194-fig6-figsupp1-data2.zip › Figure 6-figure supplement 1-source data 2/MG132_Bortezomib_Me4BoVS.tif]
