# Supplementary material for: Impairment of lipid homeostasis causes lysosomal accumulation of endogenous protein aggregates through ESCRT disruption
Source: eLife. 2024 Dec 23;12:RP86194. doi: 10.7554/eLife.86194 (PMC11666243; doi:10.7554/eLife.86194)
Supplement: Figure 3—figure supplement 2—source data 1. [file elife-86194-fig3-figsupp2-data1.pdf]

Figure 3 – figure supplement 2 – source data 1

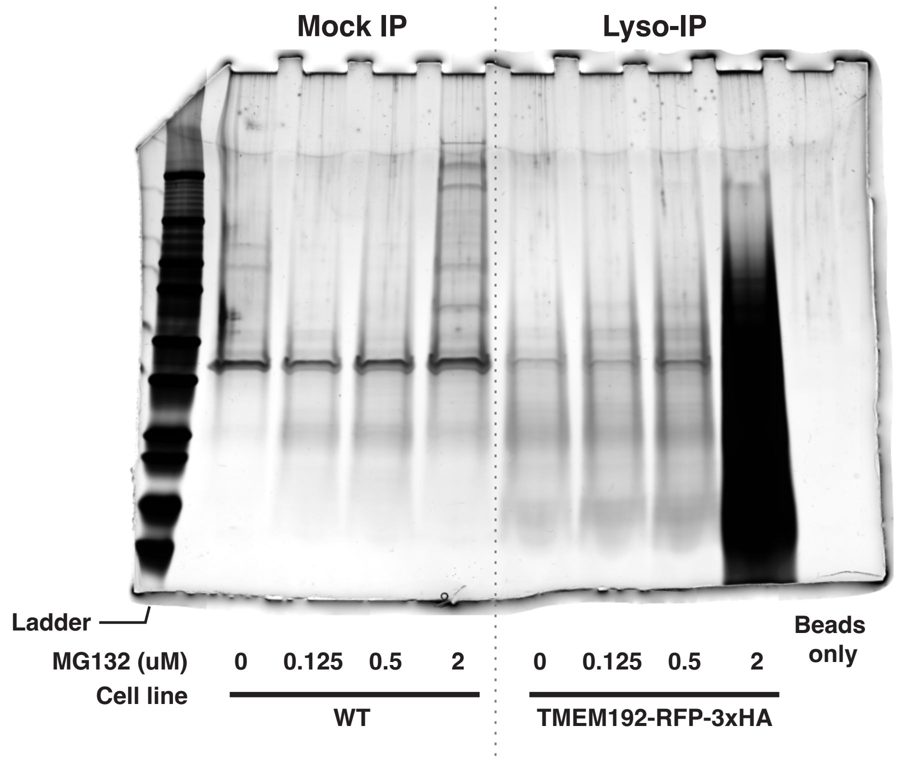

Ladder = BioRad Precision Plus Dual Color Protein Standards
