# Supplementary material for: Impairment of lipid homeostasis causes lysosomal accumulation of endogenous protein aggregates through ESCRT disruption
Source: eLife. 2024 Dec 23;12:RP86194. doi: 10.7554/eLife.86194 (PMC11666243; doi:10.7554/eLife.86194)
Supplement: Figure 6—figure supplement 1—source data 1. [file elife-86194-fig6-figsupp1-data1.pdf]

Figure 6 – figure supplement 1 – source data 1

Blue epifluorescence signal of Me4BoVS labeling before cell lysis

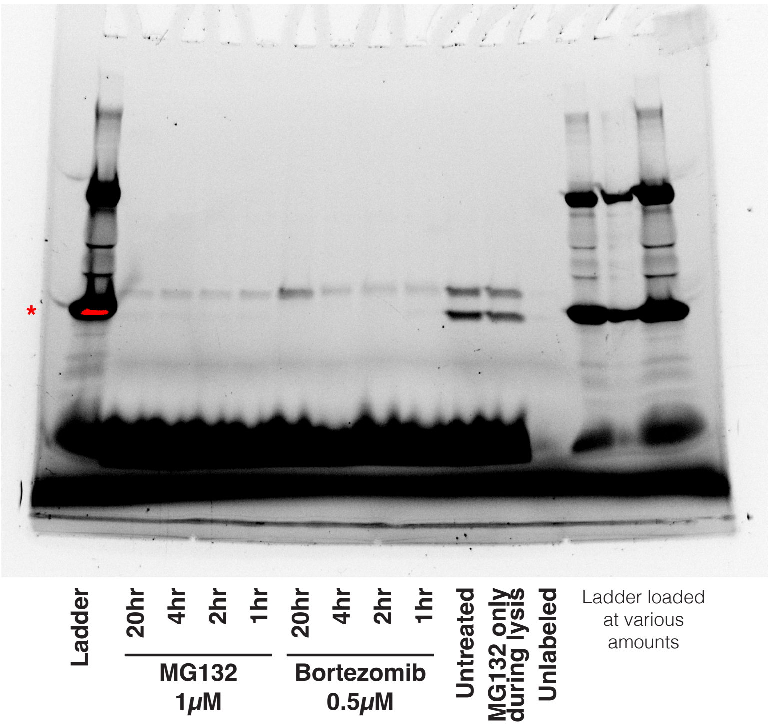

Ladder = BioRad Precision Plus Dual Color Protein Standards

* indicates signal saturation
